# Value of triglyceride glucose-body mass index in predicting nonalcoholic fatty liver disease in individuals with type 2 diabetes mellitus

**DOI:** 10.3389/fendo.2024.1425024

**Published:** 2025-01-20

**Authors:** Xiaoyi Qian, Wenwen Wu, Boyang Chen, Simin Zhang, Chunmei Xiao, Long Chen, Jun Chen, Lingli Ke, Meian He, Xiulou Li

**Affiliations:** ^1^ Department of Public Health, Hubei University of Medicine, Shiyan, Hubei, China; ^2^ Department of Nutrition and Food Hygiene, Hubei Key Laboratory of Food Nutrition and Safety, School of Public Health, Tongji Medical College, Huazhong University of Science and Technology, Wuhan, Hubei, China; ^3^ Department of Health Examination Center, Sinopharm Dongfeng General Hospital, Hubei University of Medicine, Shiyan, Hubei, China; ^4^ Department of Occupational and Environmental Health, Ministry of Education and State Key Laboratory of Environmental Health (Incubating), School of Public Health, Tongji Medical College, Huazhong University of Science and Technology, Wuhan, China

**Keywords:** type 2 diabetes mellitus, body mass index, triglyceride glucose body mass index, nonalcoholic fatty liver disease, receiver operating characteristic curve

## Abstract

**Background:**

There is limited data on the association between TyG-BMI and NAFLD in patients with Type 2 Diabetes Mellitus (T2DM). The magnitude of risk prediction and predictive efficacy of TyG-BMI for T2DM with NAFLD remains unclear.

**Objective:**

To examine the association of TyG-BMI with NAFLD in T2DM patients and assess the effectiveness of screening using the TyG-BMI index.

**Methods:**

We conducted a retrospective analysis of clinical data from 602 T2DM patients at an enterprise health lodge from September 2021 to November 2022. Patients were categorized into two groups: T2DM alone (n=250) and T2DM with NAFLD (n=352). The Mann-Whitney U test was used for comparing non-normally distributed continuous data between groups, while the Chi-square test was used for categorical data. Logistic regression analysis was performed to evaluate the effect of BMI, TyG index, and TyG-BMI index on NAFLD. The ROC curve was used to assess the predictive efficacy of the TyG-BMI index for NAFLD in T2DM patients.

**Results:**

BMI predicted the development of NAFLD in T2DM patients with an area under the receiver operating characteristic (ROC) curve of 0.792 (95% CI 0.757-0.828), and the optimal cutoff value was 25.22, with 72.2% sensitivity and 71.6% specificity; The area under the receiver operating characteristic (ROC) curve of the TyG index to predict the development of NAFLD in patients with T2DM was 0.755 (95% CI 0.716-0.794), and the optimal cutoff value was 8. 945, with a sensitivity of 80.1% and a specificity of 59.2%; The area under the receiver operating characteristic (ROC) curve of TyG-BMI index to predict the development of NAFLD in T2DM patients was 0.852, (95% CI 0.822-0.882), and the optimal cutoff value was 227.385, with a sensitivity and specificity of 80.1% and 59.2%, respectively.

**Conclusions:**

The TyG-BMI index is a significant predictor of comorbid NAFLD in T2DM patients and provides better screening performance than BMI alone. The TyG-BMI index shows promise as an early screening tool for NAFLD in T2DM patients.

## Introduction

According to the 2021 Diabetes Atlas report by the International Diabetes Federation (IDF) (1), China has the highest number of adults with diabetes. Among patients with Type 2 Diabetes Mellitus (T2DM), the incidence of Nonalcoholic Fatty Liver Disease (NAFLD) exceeds 55%, more than twice the rate in the general population (2, 3). NAFLD prevalence is high among T2DM patients, who also have a significant prevalence of obesity (up to 70%) (4) and more pronounced metabolic disorders, including glycolipid abnormalities and insulin resistance (IR).

In the early stages, T2DM with NAFLD may not present clear clinical symptoms. Common imaging diagnostic methods rely on subjective physician judgment and have limited sensitivity for detecting mild fatty liver (5). Techniques such as computed tomography (CT), magnetic resonance imaging (MRI), proton magnetic resonance spectroscopy (1H-MRS), and controlled attenuation parameters (CAP) are subject to individual variability (6, 7), and their diagnostic accuracy requires further investigation. While liver biopsy is considered the gold standard for diagnosing liver diseases, its availability and cost limit its use in routine clinical screening (8, 9). Noninvasive clinical indices that are operator-independent and not constrained by examiner variability are crucial for early screening of T2DM with NAFLD, offering simplicity and cost-effectiveness.

The triglyceride glucose index (TyG) is a convenient measure of insulin resistance. The triglyceride glucose body mass index (TyG-BMI) is a newly developed index that combines the TyG index with BMI for assessing insulin resistance. An NHANES clinical analysis (10) has shown that both TyG and TyG-BMI indexes are strongly associated with insulin resistance. The TyG index incorporates triglycerides (TG) and fasting blood glucose (FBG), while BMI includes height and weight—basic clinical indicators obtained during routine physical examinations.

However, the effects of the TyG-BMI index in the context of T2DM combined with NAFLD have not been previously reported. The risk prediction magnitude and predictive efficacy of the TyG-BMI index for T2DM combined with NAFLD remain unclear. Therefore, this study aims to provide a theoretical basis for effectively screening high-risk populations by utilizing combined indexes of TG, FBG, and BMI in T2DM patients for early detection of NAFLD.

## Methods

### Study design

This was a single-center cross-sectional study using a convenience sampling method with data derived from the electronic health physical examination file system of the health cabin of a machinery manufacturing enterprise in Hubei Province, China. Diabetic workers with complete information including baseline data and physical examination data were the subjects of this study. The health cabin is a pilot project of employee health management established in the enterprise by a first-class tertiary-level general hospital and a prevention and treatment center for occupational disease in 2019. The study followed the Declaration of Helsinki. Ethics approval was granted by the ethics committee of Hubei University of Medicine (NO. 2022-RE-033).

### Study population

The inclusion criteria for this study were patients with T2DM who had a previous diagnosis of diabetes mellitus by a health care professional or who were being treated with glucose-lowering medications Fasting blood glucose (FBG) ≥ 7.0 mmol/L or 2 h postprandial blood glucose (2hPG) ≥ 11.1 mmol/L (11). All subjects were provided with written informed consent and agreed to participate in the study. Exclusion criteria: (1) patients with diabetic ketosis, gestational diabetes mellitus, and secondary diabetes mellitus. (2) Patients with liver disease due to alcoholic, viral, drug-induced hepatitis, autoimmune, and other genetic diseases. (3) Recent excessive drinkers on exertion (alcohol intake: men > 30 g/d women > 20 g/d). (4) Had been taking medications that affect liver function and lipid levels (e.g., aspirin, sulfonamides, estrogens) for nearly 3 months. (5) Patients who had lost > 10% of their body weight in nearly 3 months as a result of taking weight-loss medication were excluded. A total of 602 T2DM patients with complete data were included in this study through the completeness check of the questionnaire and laboratory data.

### Demographic and health information

Uniformly trained investigators collected information on gender, age, marital status, cultural level, and lifestyle through the face-to-face survey. Marital status is divided into three categories: unmarried, married, and other. The educational level was classified into three categories: junior high school and below, senior high school/junior high school, and undergraduate/specialty. Lifestyle information included smoking, alcohol consumption, and physical activity (answered “yes” or “no”).

Chronic disease history was assessed by asking participants if they had a history of diabetic ketosis, gestational diabetes mellitus, and secondary diabetes mellitus.

### Anthropometric measurements

Measurements, such as weight, and height, were obtained using standard protocols. Systolic blood pressure (SBP) and diastolic blood pressure (DBP) were measured using an electronic sphygmomanometer, and the average value of the two records was taken.

### Laboratory analysis

The laboratory test results mainly include glycated hemoglobin (HbA1c), fasting blood glucose (FBG), uric acid (UA), alanine aminotransferase (ALT), aspartate aminotransferase (AST), total cholesterol (TC), triglycerides (TG), high-density lipoprotein cholesterol (HDL-C), and low-density lipoprotein cholesterol (LDL-C) levels. To accurately measure the above indicators, participants were told to fast for 8-12 hours before taking blood samples. All blood samples were stored in the refrigerator at minus 20 degrees. Measurements were performed utilizing a fully automated biochemical analyzer and an integrated biochemical and immunological machine (model: Abbott A3600, CI16200).

### Measurement of BMI

BMI = weight/(height)^2^ (weight in Kg, height in m). According to the Chinese adult weight determination criteria (12): thin and normal BMI<24.0 kg/m^2^; overweight, 24.0 kg/m^2^ ≤ BMI<28.0 kg/m^2^; obese, BMI ≥ 28.0 kg/m^2^.

### Ultrasonography

Fasting ≥ 8 hours was required before performing ultrasound examinations. The ultrasound experts uniformly reported the diagnosis results according to the criteria of the Chinese Guidelines for the Prevention and Treatment of Non-Alcoholic Fatty Liver Disease (2018 updated version) (13).

### Triglyceride glucose-body mass index

TyG-BMI index = TyG index × BMI; TyG index = Ln (TG × GLU)/2, where the unit of TG is (mg/dl) and the unit of FBG is (mg/dl). TG, 1mmol/LTG = 88.545mg/dl TG, FBG unit conversion formula is: 1mmol/LFBG = 18.02mg/dl FBG (14).

### Sample size calculation principles

This study was a cross-sectional study, and based on the results of reviewing previous survey studies, the global prevalence of T2DM combined with NAFLD was approximately 55.5%, set two-sided α= 0.05, a tolerance error of 5%, a sample size of n = 398 was calculated using PASS 15, considering the loss to follow-up rate of 20%, at least 478 T2DM patients need to be investigated.

### Statistical analysis

Data were analyzed by Spss23.0 statistical software (IBM, Armonk, NY, USA). Medians and interquartile ranges (25th-75th) were used for non-normally distributed continuous data, and comparisons of probability distributions between the subgroups were performed using the Wilcoxon rank−sum test, and multigroup comparisons were performed using the Kruskal−Wallis test. Categorical data were described as numbers and percentages (%), and the χ2 test was used for comparison between subgroups. The influencing factors of concurrent NAFLD in T2DM patients were analyzed using logistic regression. The ROC curve analysis was used to evaluate the logistic model. All statistical tests were two-sided and the p-value of 0.05 was considered statistically significant.

## Results

### Clinical characteristic

**Table 1** shows the basic characteristics of all participants. A total of 602 patients with T2DM were included, including 568 males and 34 females; The median age was 49 years; The number of T2DM patients with concurrent NAFLD was 352, with a detection rate of approximately 58.5%. The prevalence of NAFLD in diabetic patients with different genders, ages, marital statuses, and exercises was statistically significant (all *p* < 0.05). There were significant differences in SBP, DBP, HbA1c, FBG, UA, alt, AST, TC, TG, LDL-C, BMI, TyG index, and TyG-BMI index between the T2DM group with NAFLD and the pure T2DM group (all *p* < 0.05). HDL-C was lower in the group of T2DM comorbid NAFLD than in the T2DM group (*p* < 0.05). There were no significant differences in culture level, smoking status, or drinking status between the two groups (*p* > 0.05).

**Table 1:** Comparison of BMI, TyG index, TyG-BMI index, and biochemical indexes between T2DM patients and those complicated with NAFLD.

Variables	Number of (n=602)	T2DM (n=250)	T2DM with NAFLD (n=352)	*χ2/Z* Value	*p* Value
Sex				10.124	0.001
Male	568 (94.4)	227 (90.8)	341 (96.4)		
Female	34 (5.6)	23 (9.2)	11 (3.1)		
Age^b^	49 (46,53)	49 (46,53)	49 (46,52)	-2.121	0.034
Marital Status				6.527	0.038
Unmarried	188 (31.2)	92 (36.8)	96 (27.3)		
Married	359 (59.6)	135 (54.0)	224 (63.6)		
Others	55 (9.1)	23 (9.2)	32 (9.1)		
Educational Level ^a^				5.676	0.06
Junior High School or below	67 (11.1)	24 (9.6)	43 (12.2)		
Junior High School/Technical Secondary School	356 (59.1)	162 (64.8)	194 (55.1)		
Undergraduate/Junior College	179 (29.7)	64 (25.6)	115 (32.7)		
Smoking				0.135	0.713
No	328 (54.5)	134 (53.6)	194 (55.1)		
Yes	274 (45.5)	116 (46.4)	158 (44.9)		
Drinking				0.112	0.738
No	354 (58.8)	149 (59.6)	205 (58.2)		
Yes	248 (41.2)	65 (41.9)	147 (41.8)		
Lack of exercise				4.290	0.038
No	271 (45.0)	125 (50.0)	146 (41.5)		
Yes	331 (55.0)	125 (50.0)	206 (58.5)		
SBP (mm Hg) ^b^	128 (117,141)	121 (112,134)	133 (122,146.75)	-7.445	<0.001
DBP (mm Hg) ^b^	80 (73,90)	76 (69,83)	83.5 (76,93.75)	-7.754	<0.001
HbA1c (%) ^b^	6.5 (5.8,7.6)	6.2 (5.5,7.2)	6.8 (6.0,7.7)	-4.467	<0.001
FBG (mmol/L) ^b^	7.1 (6,8.9)	6.6 (5.6,8.2)	7.4 (6.4,9.3)	-5.023	<0.001
UA (μmol/L) ^b^	364 (301,432)	337 (284,395)	381.5 (316,454)	-5.092	<0.001
ALT (μ/L) ^b^	24 (16,36)	19 (13,28)	28 (19,42)	-8.389	<0.001
AST (μ/L) ^b^	27 (22,34)	23 (20,29)	29 (23,37)	-6.692	<0.001
TC (mmol/L) ^b^	4.65 (4.04,5.42)	4.43 (3.80,5.17)	4.88 (4.25,5.62)	-5.557	<0.001
TG (mmol/L) ^b^	1.60 (1.06,2.6)	1.12 (0.8,1.64)	2.04 (1.40,3.05)	-10.863	<0.001
HDL-C (mmol/L) ^b^	1.01 (0.88,1.17)	1.11 (0.94,1.28)	0.97 (0.84,1.10)	-6.790	<0.001
LDL-C (mmol/L) ^b^	2.73 (2.12,3.35)	2.60 (2.11,3.17)	2.83 (2.16,3.47)	-2.737	0.006
BMI (Kg/m^2^) ^b^	25.46 (23.38,27.66)	23.67 (22.17,25.40)	26.90 (24.94,29.07)	-12.238	<0.001
TyG index ^b^	9.16 (8.72,9.75)	8.82 (8.36,9.21)	9.45 (9.00,10.00)	-10.661	<0.001
TyG-BMI index ^b^	235.15 (209.31,263.86)	209.93 (189.51,228.17)	255.43 (231.70,280.46)	-14.729	<0.001

BMI, body mass index; SBP, systolic blood pressure; DBP, diastolic blood pressure; HbA1c, glycosylated hemoglobin; FBG, fasting blood glucose; UA, uric acid; ALT, alanine aminotransferase; AST, aspartate aminotransferase; TC, total cholesterol; TG, triglyceride; HDL-C, high-density lipoprotein cholesterol; LDL-C, low-density lipoprotein cholesterol ^a^n (%), ^b^M (P_25_, P_75_).

### Logistic regression analysis of potential factors of NAFLD in T2DM patients

Whether T2DM patients with NAFLD or not were used as the dependent variable, BMI, TyG index, and TyG-BMI index were included as independent variables, Multivariate analysis was performed after adjusting for gender, age, marital status, exercise status, SBP, DBP, HbA1c, FBG, UA, alt, AST, TC, TG, LDL-C which were statistically different in univariate, The results showed(**Table 2**) that BMI, TyG index, and TyG-BMI index were associated with T2DM and risk factors for NAFLD (p > 0.05).

**Table 2:** Logistic regression analysis of influencing factors of T2DM combined with NAFLD.

	Variables	*β*	*Waldχ2*	*OR*	95%*CI*	*p* Value
Before adjustment	BMI	0.437	112.057	1.548	1.428-1.679	<0.001
	TyG index	1.388	88.739	4.008	3.003-5.350	<0.001
	TyG-BMI index	0.048	138.064	1.049	1.041-1.058	<0.001
After adjusting for	BMI	0.362	47.29	1.436	1.295-1.592	<0.001
	TyG index	1.741	15.699	5.704	2.411-13.496	<0.001
	TyG-BMI index	0.042	52.479	1.042	1.031-1.054	<0.001

### ROC curves to predict BMI, TyG index, and TyG-BMI indices for the risk of comorbid NAFLD in patients with T2DM

**Figure 1:**
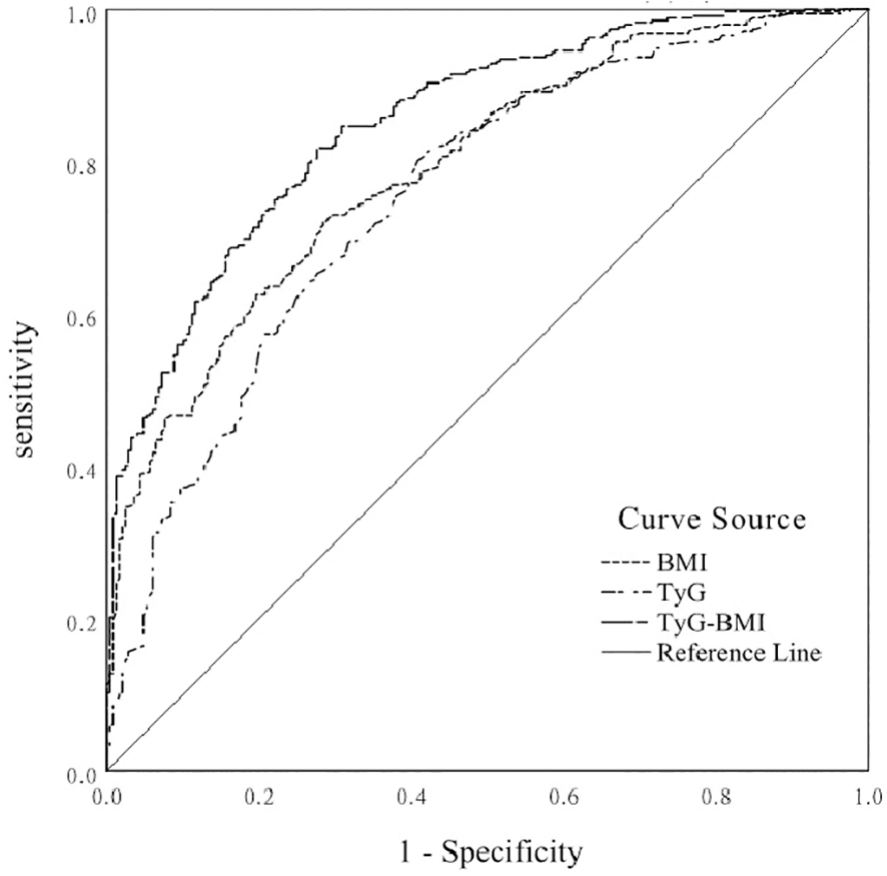
ROC curve of BMI, TyG index, and TyG-BMI index for predicting the risk of NAFLD in T2DM patients.

Considering T2DM patients with NAFLD as a positive diagnosis, the results of ROC curve analysis and AUC and their corresponding 95% CIs for BMI, TyG index, and TyG-BMI index are shown in **Figure 1**. TyG-BMI index showed the largest AUC (0.852, 95% CI 0.822-0.882), followed by the BMI (0.792, 95% CI 0.757-0.828) and TyG index (0.755, 95% CI 0.716-0.794) in all subjects (*p* < 0.001). BMI had a diagnostic cut-off value of 25.22, with a sensitivity of 72.2%, and a specificity of 71.6% for T2DM patients with NAFLD. TyG index had a diagnostic cut-off value of 8. 945, a sensitivity of 80.1%, and a specificity of 59.2% for T2DM patients with NAFLD. TyG-BMI index had a diagnostic cut-off value of 227.385, a sensitivity of 81.8%, and a specificity of 72.4% for T2DM patients with NAFLD. The results indicated that the diagnostic effect of the TyG-BMI index was better than that of other parameters, and the TyG-BMI index was characterized by its potential clinical value for T2DM patients with NAFLD.

## Discussion

The recent changes in the prevalence of NAFLD parallel the epidemic trends of obesity and T2DM, and the prevalence of T2DM patients with NAFLD has increased year by year. Previous studies have shown that approximately 57% - 80% of patients with T2DM had concurrent NAFLD (15, 16). Chinese expert consensus proposed that clinical screening for T2DM patients with NAFLD should be given full attention (13). In this study, we initially investigated the evaluation and predictive value of BMI, TyG index, and TyG-BMI index on the risk of NAFLD in T2DM patients. The prevalence of NAFLD among T2DM patients in the machinery manufacturer was 62.0%, which was similar to the results of Dong et al. (17). This study showed that the glycolipid metabolism disorder was more obvious in the group of T2DM comorbid NAFLD than in the T2DM group, and a higher prevalence level of NAFLD should be paid more attention by enterprise managers. Other studies have shown that if people have T2DM comorbid NAFLD, the risk of metabolic abnormalities, extrahepatic target organ damage, and cardiovascular complications would gradually increase, which may further promote the progression of NAFLD-related hepatitis and cirrhosis and increase the risk of developing end-stage liver disease (18–20). If the health of workers is guaranteed, it will greatly improve production efficiency and reduce the corporate medical burden.

BMI is currently one of the most commonly used measures to judge a healthy weight. Studies have implicated obesity and IR as a shared pathogenesis of T2DM with NAFLD. Zhai MX and other studies found that the prevalence of comorbid NAFLD in patients with T2DM increased with increasing BMI, the elevation was most pronounced when BMI was ≥ 25 kg/m^2^ (18). The results of relevant studies similarly (17–20) suggested that BMI or obesity was an important risk factor for T2DM combined with NAFLD (21–23). Consider the reason for this as overweight/obesity and IR through body compensation and lipolysis, forcing lipid transfer to hepatocytes and accumulation, abnormal lipid metabolism allows a sustained increase in free fatty acids in the body, impedes normal insulin secretion and IR. Patients with T2DM have increased levels of free unsaturated fatty acids in the circulation and liver caused by disturbed glycolipid metabolism, excess free fatty acids are converted into lipids intrahepatic-ally (24), and multiple factors contribute to hepatic steatosis, further inducing the formation of NAFLD.

The TyG index, a novel index proposed by Simental - mendía in 2008(14), has been recognized as a reliable marker for IR (25). The TyG-BMI index incorporates lipid, glucose, and adiposity measures. While predicting NAFLD, TyG-BMI can also assess components of the metabolic syndrome, which consists of obesity, dyslipidemia, and measures of glycemia. This study showed that TyG-BMI was a contributing factor to NAFLD in T2DM patients. Meanwhile, the results of ROC curve analysis suggested that TyG-BMI had a better value for predicting NAFLD in T2DM patients. Zhang et al. (26) found that the TyG-BMI index was the best predictor of prediabetes in adults, and the risk of T2DM patients with NAFLD increased by 2 times for every 1 SD increase in the TyG-BMI index. Based on the previous findings, it was speculated that IR may be mainly mediating the association. Studies have shown that IR can promote the development of NAFLD by inducing the increased breakdown of adipose tissue TG and *de novo* synthesis of intrahepatic TG (18, 27). IR was closely related to islet function, which was induced by elevated blood glucose and lipid-impaired - cell secretory function, the body’s compensatory ability to secrete insulin to maintain glucose metabolism was reduced, the inhibition of lipolysis was also reduced, interference with the liver’s normal metabolism of glycolipids, excessive free fatty acids are deposited in hepatocytes. Sustained IR manifests as high glucolipotoxicity, which can further lead to cellular stress responses, such as oxidative stress, endoplasmic reticulum stress, and lipid peroxidation. Under this environment, mitochondrial dysmetabolism of hepatocytes, and IR activates hepatic stellate cells and macrophages to secrete inflammatory factors and mediate the occurrence of adverse outcomes in liver disease (28, 29). NAFLD has a higher incidence in patients with T2DM. Combining BMI with the TyG index as a composite index of the TyG-BMI index may improve diagnostic efficacy. Composite parameters that allow for early screening and management of abnormalities may help minimize morbidity in T2DM patients with NAFLD.

### Strengths and limitations

The innovation of this study lies in evaluating the relationship between BMI, TyG index, and TyG-BMI index with NAFLD in T2DM patients, and finding that the combination of TyG-BMI index with BMI has a significant effect on NAFLD in T2DM patients, with diagnostic value superior to BMI alone. However, an important limitation of this study is that the majority of participants are male, which may impact the generalizability of the results. Due to the imbalance in gender distribution, the findings may differ in female populations. Therefore, future research should validate the effectiveness of the TyG-BMI index in more diverse samples, particularly in female patients.

Currently, the combination of BMI and TyG-BMI index shows good results in screening for NAFLD in T2DM patients. To further validate these findings and address the limitations of the current study, we suggest conducting large-scale cohort studies in the future to confirm the diagnostic value of these indicators across different genders and populations.

## Data Availability

The original contributions presented in the study are included in the article/supplementary material. Further inquiries can be directed to the corresponding author/s.

## References

[1] Sun H Saeedi P Karuranga S Pinkepank M Ogurtsova K Duncan BB. IDF Diabetes Atlas: Global, regional, and country-level diabetes prevalence estimates for 2021 and projections for 2045. Diabetes Res Clin Practice. (2022) 183:109–19. doi: 10.1016/j.diabres.2021.109119 PMC11057359 34879977

[2] Xia M Sun X Zheng L Bi Y Li Q Sun L. Regional difference in the susceptibility of non-alcoholic fatty liver disease in China. BMJ Open Diabetes Care. (2020) 8:e001311. doi: 10.1136/bmjdrc-2020-001311 PMC7287499 32522731

[3] Younossi ZM Golabi P de Avila L Paik JM Srishord M Fukui N. The global epidemiology of NAFLD and NASH in patients with type 2 diabetes: A systematic review and meta-analysis. J Hepatol. (2019) 71:793–801. doi: 10.1016/j.jhep.2019.06.021 31279902

[4] Mandal A Bhattarai B Kafle P Khalid M Jonnadula SK Lamicchane J. Elevated liver enzymes in patients with type 2 diabetes mellitus and non-alcoholic fatty liver disease. Cureus. (2018) 10:e3626. doi: 10.7759/cureus.3626 30697502 PMC6347442

[5] Jia H Yiwen S Xiaoning Wu. Application value of imaging diagnosis in nonalcoholic fatty liver disease. J Clin Hepatol. (2018) 34:2698–701. doi: 10.3969/j.issn.1001-5256.2018.12.011

[6] Du J Yang Z. Advances in the use of imaging in liver fat quantification. Radiologic Pract. (2017) 32:479–82. doi: 10.3760/cma.j.issn.1001-6180.2017.06.009

[7] Selvaraj EA Mózes FE Jayaswal ANA Zafarmand MH Vali Y Lee JA. Diagnostic accuracy of elastography and magnetic resonance imaging in patients with NAFLD: A systematic review and meta-analysis. J Hepatol. (2021) 75:770–85. doi: 10.1016/j.jhep.2021.04.044 33991635

[8] Chinese Society of Endocrinology Chinese Diabetes Society. Management of Chinese adults with type 2 diabetes and non-alcoholic fatty liver disease: an expert consensus. Chin J Endocrinol Metab. (2021) 37:589–98. doi: 10.3760/cma.j.cn112138-20210105-00058

[9] Lee BW Lee YH Park CY Rhee EJ Lee WY Kim NH. Non-alcoholic fatty liver disease in patients with type 2 diabetes mellitus: A position statement of the fatty liver research group of the Korean diabetes association. Diabetes Metab J. (2020) 44:382–401. doi: 10.4093/dmj.2020.0010 32431115 PMC7332334

[10] Lim J Kim J Koo SH Kwon GC. Comparison of triglyceride glucose index, and related parameters to predict insulin resistance in Korean adults: An analysis of the 2007-2010 Korean National Health and Nutrition Examination Survey. PloS One. (2019) 14:e0212963. doi: 10.1371/journal.pone.0212963 30845237 PMC6405083

[11] Alberti GK Zimmet ZP. Definition, diagnosis and classification of diabetes mellitus and its complications. Part 1: diagnosis and classification of diabetes mellitus provisional report of a WHO consultation. Diabetic medicine: J Br Diabetic Assoc. (1998) 15:539. doi: 10.1002/(SICI)1096-9136(199807)15:7<539::AID-DIA668>3.0.CO;2-S 9686693

[12] Chen C Lu FC. Guidelines for the prevention and control of overweight and obesity in chinese adults (excerpt). *China working group on obesity* . Acta Nutrimental Sinical. (2004) 1:1–4. doi: 10.3321/j.issn:1001-9844.2004.01.002 15807475

[13] National Workshop on Fatty Liver and Alcoholic Liver Disease Chinese Society of Hepatology Chinese Medical Association Fatty Liver Expert Committee Chinese Medical Doctor Association. Guidelines of prevention and treatment for nonalcoholic fatty liver disease: a 2018 update. J Pract Hepatol. (2018) 34:947–57. doi: 10.3969/j.issn.1005-3277.2018.10.001

[14] Simental-Mendía LE Rodríguez-Morán M Guerrero-Romero F. The product of fasting glucose and triglycerides as a surrogate for identifying insulin resistance in apparently healthy subjects. Metab Syndr Relat Disord. (2008) 6:299–304. doi: 10.1089/met.2008.0014 19067533

[15] Chinese Medical Association Endocrinology Branch. Consensus for diagnosis and treatment of nonalcoholic fatty liver diseases and metabolic disorders (2nd Edition). J Clin Hepatol. (2018) 34:2103–8. doi: 10.3969/j.issn.1001-5256.2018.12.003

[16] Stefan N Cusi K. A global view of the interplay between non-alcoholic fatty liver disease and diabetes. Lancet Diabetes Endocrinol. (2022) 10:284–96. doi: 10.1016/S2213-8587(22)00003-1 35183303

[17] Dong C Wu F Li X He L Gao S Li X. Correlation of alanine aminotransferase and carotid atherosclerosis in type 2 diabetic patients with non-alcoholic fatty liver disease. J Xi’an Jiao Tong University: Med Edition. (2013) 34:512–5. doi: 10.7518/j.issn.1001-9468.2013.03.012

[18] Li T Bai X. Two-way relationship and pathogenesis of nonalcoholic fatty liver disease and type 2 diabetes mellitus. Med Recapitulate. (2021) 27:158–162,168. doi: 10.3969/j.issn.1000-2737.2021.02.012

[19] Targher G Corey KE Byrne CD Roden M. The complex link between NAFLD and type 2 diabetes mellitus - mechanisms and treatments. Nat Rev Gastroenterol Hepatol. (2021) 18:599–612. doi: 10.1038/s41575-021-00448-y 33972770

[20] Gao X Wang S. Advancement of research on the association of non-alcoholic fatty liver disease with type 2 diabetes mellitus. Chin J Hepatology. (2014) 22:161–4. doi: 10.3760/cma.j.issn.1000-9991.2014.02.013 24919218

[21] Ren R. Risk factors of nonalcoholic fatty liver disease in patients with T2DM. Shanxi Med J. (2019) 48:704–5. doi: 10.3969/j.issn.1003-9787.2019.04.017

[22] Sun X. Analysis of factors associated with type 2 diabetes mellitus combined with non-alcoholic fatty liver disease. Chin Prev Med. (2019) 20:78–80. doi: 10.3969/j.issn.1002-2773.2019.01.020

[23] Wu T Yin S Wang H Zhang H Jia W Ji G. The clinical features and risk factors of a patient with Metab.olic syndrome combined with non-alcoholic fatty liver disease. J Clin Hepatol. (2011) 27:1036–40. doi: 10.3969/j.issn.1001-5256.2011.12.020

[24] Zwartjes MSZ Gerdes VEA Nieuwdorp M. The role of gut microbiota and its produced metabolites in obesity, dyslipidemia, adipocyte dysfunction, and its interventions. Metabolites. (2021) 11:531. doi: 10.3390/metabo11080531 34436472 PMC8398981

[25] Won K-B Park G-M Lee S-E Cho I-J Kim HC Lee BK. Relationship of insulin resistance estimated by triglyceride glucose index to arterial stiffness. Lipids Health Dis. (2018) 17:268. doi: 10.1186/s12944-018-0914-2 30474549 PMC6260653

[26] Zhang S Du T Li M Jia J Lu H Lin X. Triglyceride glucose-body mass index is effective in identifying nonalcoholic fatty liver disease in nonobese subjects. Med (Baltimore). (2017) 96:e7041. doi: 10.1097/MD.0000000000007041 PMC5459725 28562560

[27] Samuel VT Shulman GI. The pathogenesis of insulin resistance: integrating signaling pathways and substrate flux. J Clin Invest. (2016) 126:12–22. doi: 10.1172/JCI77812 26727229 PMC4701542

[28] Yan L Zuo J Wu M Tong H. Progress of the research on the relationship between fatty acid metabolism and nonalcoholic fatty liver disease. China Med And Pharmacy. (2020) 10:35–39,56. doi: 10.1172/JCI84203

[29] Liu J Ren C Feng Y He J. Relationship between liver fibrosis and insulin resistance in patients with type 2 diabetes and nonalcoholic fatty liver disease. Chin J Clin Res. (2021) 34:443–8. doi: 10.3969/j.issn.2095-6114.2020.02.006

